# Whom should we target? A brief report on a prospective study to identify predictors of mental health and self-care worsening in patients with diabetes mellitus during the COVID-19 pandemic

**DOI:** 10.20945/2359-4292-2023-0073

**Published:** 2024-08-05

**Authors:** Janine Alessi, Isadora Nunes Erthal, Julia Belato Teixeira, Beatriz D. Schaan, Gabriela H. Telo

**Affiliations:** 1 Serviço de Medicina Interna Hospital São Lucas Pontifícia Universidade Católica do Rio Grande do Sul Porto Alegre RS Brasil Serviço de Medicina Interna, Hospital São Lucas, Pontifícia Universidade Católica do Rio Grande do Sul, Porto Alegre, RS, Brasil; 2 Faculdade de Medicina Pontifícia Universidade Católica do Rio Grande do Sul Porto Alegre RS Brasil Faculdade de Medicina, Pontifícia Universidade Católica do Rio Grande do Sul, Porto Alegre, RS, Brasil; 3 Serviço de Endocrinologia Hospital de Clínicas de Porto Alegre Porto Alegre RS Brasil Serviço de Endocrinologia, Hospital de Clínicas de Porto Alegre, Porto Alegre, RS, Brasil; 4 Faculdade de Medicina Universidade Federal do Rio Grande do Sul Porto Alegre RS Brasil Faculdade de Medicina, Universidade Federal do Rio Grande do Sul, Porto Alegre, RS, Brasil; 5 Programa de Pós-graduação em Medicina e Ciências da Saúde Pontifícia Universidade Católica do Rio Grande do Sul Porto Alegre RS Brasil Programa de Pós-graduação em Medicina e Ciências da Saúde, Pontifícia Universidade Católica do Rio Grande do Sul, Porto Alegre, RS, Brasil

**Keywords:** Depression, anxiety, outbreak, health care, diabetes

## Abstract

**Objective:**

To identify predictors of mental health disorders and self-care worsening in patients with diabetes through 15 months of COVID-19 pandemic.

**Subjects and methods:**

Prospective study following patients with type 1 and type 2 diabetes during the COVID-19 pandemic in Southern Brazil. Participants were evaluated through phone calls in two moments: first three months of the outbreak, and 15 months later. The outcomes were the assessment of worsening in mental health disorders (increase ≥ 10% in the total score of the Self-Report Questionnaire), the assessment of emotional distress related to diabetes (increase ≥ 10% in the total score of the Brazilian version of the Problem Areas in Diabetes), and worsening in self-care parameters (reduction ≥ 3 points in the Self-Care Inventory-Revised). Logistic regression models were used to determine the odds ratio (OR) and their respective confidence intervals. Point-biserial correlation coefficients (r^pb^) were used to measure the relationship between the variation in scores and patient characteristics.

**Results:**

In total, 150 adults were enrolled (54.6 ± 13.9 years old, 58.7% female, 85.9% white), out of which 118 remained during follow up. After 18 months, 34,7% of them (52.2 ± 14.8 years old, 53.7% female, 87.5% white) worsened mental health scores. An increase in mental health disorders was experienced by patients with lower middle-income [OR 4.2 (1.2-15.0)], and those who reported greater difficulty managing diabetes [OR 3.2 (1.4-7.1); r^pb^ 0.32, P < 0.01]. In contrast, those who perceived an improvement in diabetes control showed a reduction in their mental health scores [OR 0.3 (0.1-0.8)]. For self-care, there was a score worsening in patients with longer duration of diabetes [OR 1.1 (1.0-1.1)] and in those using insulin [OR 8.3 (1.7-41.4); r^pb^ 0.23, P = 0.01]. Conversely, those who followed the social distancing guidance had an improvement in self-care [OR 0.4 (0.1-0.9); r^pb^ 0.18, P = 0.05].

**Conclusion:**

Some clinical and socioeconomic characteristics may be suitable for identifying patients at higher risk of mental health and self-care worsening, signaling who needs to be monitored more closely during crisis situations.

## INTRODUCTION

Diabetes mellitus is a long-term progressive disease that can pose emotional, social, and economic challenges. Studies have shown that people with diabetes are up to four times more likely to be affected by depression and anxiety compared to their peers without diabetes (1). Numerically, mental health disorders are about as prevalent as direct complications from diabetes, highlighting the significance of incorporating emotional assessments into standard diabetes care (2).

The coronavirus 2019 (COVID-19) pandemic has had huge impact for individuals with chronic diseases, including diabetes. The disruption of medical appointments, associated with the changes in lifestyle routines that accompanied the pandemic, negatively impacted the emotional well-being, self-care and adherence to treatment (3,4). Furthermore, the experienced stressogenic environment represented a risk factor for the exacerbation of preexisting psychiatric disorders, which is corroborated by the alarming finding that one in ten patients with diabetes had suicidal thoughts after one year of the COVID-19 pandemic (5).

Studies indicate that the COVID-19 pandemic has resulted in a range of emotional issues in individuals with diabetes (3). However, who are those who would benefit most from protective measures to prevent a decline in mental health and self-care? Our study aimed to identify factors predicting the deterioration of mental health and self-care among patients with diabetes over the 15 months of the COVID-19 pandemic.

## METHODS

This is a prospective study focused on patients with diabetes mellitus during the COVID-19 pandemic in Southern Brazil. We identified adults previously diagnosed with either type 1 or type 2 diabetes from electronic medical records. The patients were regularly monitored at one of two public tertiary care hospitals in Southern Brazil. Inclusion criteria were being aged 18 years or older, having an updated phone number in their medical records and a recent HbA1c assessment. Eligible patients were contacted in April 2020 to invite them to take part in the study, submit their informed consent, and their initial assessment was carried out. Then, in July 2021 – 15 months after their inclusion – participants were contacted again for a reassessment.

Electronic medical records were used to assess demographics and clinical characteristics, presence of diabetes complications, and previous laboratory tests. Directive questions were used to access about psychosocial aspects, use of medication, and lifestyle habits. For the evaluation of outcomes related to mental health and adherence to treatment, specific questionnaires validated for the Brazilian population were used. For the assessment of mental health disorders, the Self-Report Questionnaire (SRQ 20) was used, which consisted of 20 questions with “yes and no” answer options. For the assessment of emotional distress related to diabetes, the Brazilian version of the Problem Areas in Diabetes (B-PAID) was used. This is a 20-item questionnaire on a 4-point response scale, with responses ranging from 0 (“it is not a problem”) to 4 (“it is a serious problem”). For self-care and treatment adherence evaluation, the Self-Care Inventory-Revised (SCI-R) was used, a 14-question survey that reflects how patients with diabetes have adhered to treatment recommendations. Higher scores indicate better adherence.

The study outcomes were the assessment of predictors of: (1) worsening in mental health disorders, defined as an increase ≥ 10% in the total score of the SRQ-20 questionnaire; (2) worsening in emotional distress related to diabetes, defined as an increase ≥ 10% in the total score of the BPAID; (3) worsening in self-care parameters, defined as reduction ≥ 3 points in the SCI-R. To increase the specificity of our analysis, we pre-specified a 10% margin of tolerance in the total scores in order to consider only clinically relevant changes. The 10% margin was defined arbitrarily before the analysis, and it is equivalent to approximately 3 points in the SCI-R score.

Baseline characteristics are presented as mean ± standard deviation (SD) and percentages, and groups were compared using the chi-square (χ^2^) test for categorical variables and Student’s t-test for continuous variables. Clinical parameters and social aspects potentially modified during the pandemic were tested to identify its association with worsening in mental health, emotional distress and self-care parameters. Logistic regression models were used to determine the odds ratio (OR) and their respective 95% confidence intervals (CI). Sensitivity analysis included a model with correction for sex, age and duration of diabetes. Lastly, point-biserial correlation coefficients (*r*^pb^) were used to measure the relationship between the variation in total scores of the questionnaires and patient characteristics. An α level of ≤ 0.05 was used to determine statistical significance.

## RESULTS

**Figure 1:**
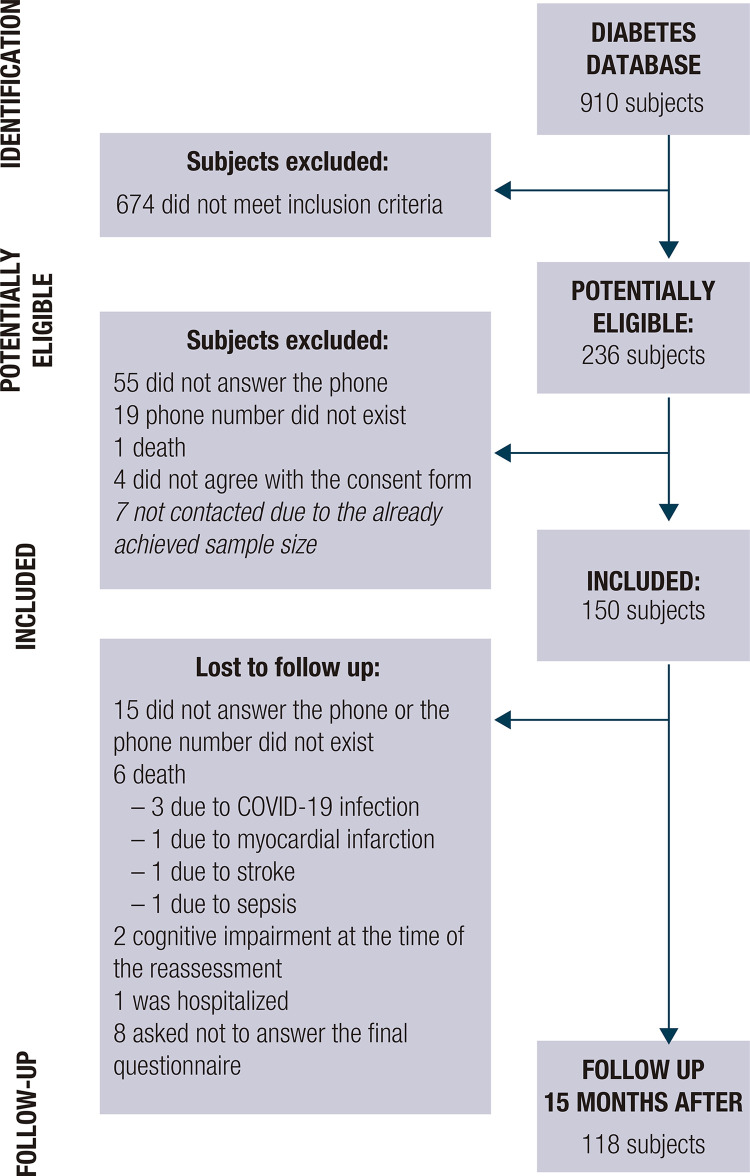
Flowchart of participant selection and baseline characteristics of the study participants.

In total, 150 adults were enrolled (54.6 ± 13.9 years old, 58.7% female, 85.9% white), out of which 118 remained during follow up (Figure 1). The characteristics of participants are described in Table 1. After 15 months, 34,7% of them (52.2 ± 14.8 years old, 53.7% female, 87.5% white) worsened mental health scores; compared to those who did not worsen, both groups had similar age, sex, skin color, duration of diabetes and HbA1c at baseline.

**Table 1:** Demographics and clinical characteristics of study participants

	Total (n = 118)	Type 1 diabetes (n = 58)	Type 2 diabetes (n = 60)
Age (years)	54.2 ± 14.0	53.6 ± 13.1	54.7 ± 14.8
Sex (% female)	56.8%	60.3%	53.3%
Self-reported skin color (% white)	89.3%	89.5%	89.1%
Diabetes duration (years)	22.1 ± 10.7	20.9 ± 11.4	23.2 ± 9.9
HbA1c (%)	8.7 ± 1.5	8.8 ± 1.6	8.7 ± 1.5
Diabetes complications			
Retinopathy	47.5%	50.0%	45.0%
Neuropathy	29.9%	22.8%	36.7%
Nephropathy	42.4%	31.0%	53.3%
Insulin use	92.5%	100%	86.8%
Metformin use	46.7%	-	46.7%
Glibenclamide use	10.0%	-	10.7%
Systemic arterial hypertension	59.3%	58.6%	60.0%
Coronary artery disease	18.6%	20.7%	16.7%
ACE inhibitors use	40.2%	49.1%	31.7%
ARB use	25.6%	21.1%	30.0%

Data are mean ± standard deviation or %. An α ≤ 0.05 indicates significant difference. HbA1c: hemoglobin A1c. ACE: Angiotensin-converting enzyme; ARB: angiotensin receptor blockers.

For mental health evaluation (SRQ-20), a worsening in mental health scores was experienced by patients with lower middle-income [OR 4.1 (1.2-15.0)], and those who reported greater difficulty in managing diabetes [OR 3.2 (1.4-7.1); *r* 0.32, P < 0.01]. In contrast, those who perceived an improvement in diabetes control showed a reduction in their mental health scores [OR 0.3 (0.1-0.8)]. Sensitivity analysis showed that these associations remained similar when the model was corrected for age, sex, and duration of diabetes (Table 2).

**Table 2:** Assessment of predictors of mental health, emotional distress and self-care worsening in patients with diabetes mellitus during the 15 months of follow up

	Worsening in mental health score (SRQ 20)	Worsening in emotional distress related to diabetes (BPAID)	Worsening in self-care (SCI-R)
Type 1 diabetes	0.8 (0.4-1.8)	1.1 (0.5-2.3)	1.0 (0.5-2.2)
Type 2 diabetes	1.2 (0.6-2.5)	0.9 (0.4-1.9)	0.9 (0.6-2.1)
Duration of diabetes	1.0 (0.9-1.0)	1.0 (0.9-1.1)	**1.1 ( 1.0-1.1)**
Baseline HbA1c	0.9 (0.7-1.1)	1.0 (0.8-1.3)	0.9 (0.7-1.2)
Family income	**4.1 (1.2-15.0)**	0.6 (0.2-2.3)	1.2 (0.4-3.7)
Insulin use	1.3 (0.3-4.8)	5.5 (0.7-45.1)	**8.3 (1.7-41.4)**
Followed the social distancing guidance	1.0 (0.4-2.3)	0.7 (0.3-1.8)	**0.4 (0.1-0.9)**
COVID-19 infection	1.1 (0.2-6.4)	1.8 (0.3-12.3)	1.4 (0.2-8.7)
Loss of a loved one	1.6 (0.7-3.7)	1.4 (0.6-3.2)	1.2 (0.5-2.8)
Job loss	1.5 (0.5-4.5)	1.5 (0.5-4.3)	1.4 (0.5-4.1)
Perceived difficulty managing diabetes	**3.2 (1.4-7.1)**	1.8 (0.8-4.0)	0.8 (0.3-1.7)
Perceived improvement in glycemic control	**0.3 (0.1-0.8)**	0.5 (0.2-1.2)	0.7 (0.4-2.7)

Data are odds ratio (OR) and 95% confidence interval (CI). HbA1c: glycated hemoglobin.

The assessment of emotional distress related to diabetes (BPAID) was not associated with any of the clinical and social parameters evaluated. Furthermore, type of diabetes, HbA1c value, having had COVID-19 infection, dealing with the loss of a loved one, or job loss during the pandemic were not associated with any of the outcomes assessed (Table 2).

For self-care assessment (SCI-R), there was a score worsening in patients with longer duration of diabetes [OR 1.1 (1.0-1.1)] and in those using insulin [OR 8.3 (1.7-41.4); *r* 0.23, P = 0.01]. Conversely, those who followed the social distancing guidance had an improvement in self-care [OR 0.4 (0.1-0.9); *r* 0.18, P = 0.05]. These associations remained similar in the corrected model (Table 2).

## DISCUSSION

This study aimed to identify predictors of mental health disorders, emotional distress, and worsening self-care habits in individuals with diabetes during the COVID-19 pandemic. Our findings show that patients of low-to-middle-income status and those struggling with diabetes management are most susceptible to declining mental health scores during crises. Regarding self-care, patients with a longer history of diabetes, especially those using insulin, appear most at risk of worsening self-care habits. These individuals could greatly benefit from protective measures aimed at mental health and self-care in similar situations in the future.

The association between family income and deteriorated mental health in individuals without diabetes has been long established (6). This linkage is primarily explained by two mechanisms: the social causation hypothesis and the health selection hypothesis. The first suggests that stress, social adversity, and reduced coping capacity associated with low income escalate the risk of emotional disorder development. The second theory posits that individuals with mental disorders might be predisposed to reduced educational attainment and lower income (6,7). Additionally to these mechanisms, the economic crisis that accompanied the COVID-19 pandemic was a trigger for the reduction of family income worldwide. A previous study showed that COVID-19 had a significantly greater negative impact among the low-income patients on family income/employment, access to food, access to mental health treatment, and family conflicts (8). For patients with diabetes mellitus, the pandemic represented an additional challenge, making it difficult to access medical care and supplies for diabetes treatment, conditions that may have contributed to individuals in greater economic vulnerability being more affected by mental health disorders.

Routine changes that accompanied the COVID-19 pandemic directly interfered with diabetes and health care, as supported by several studies. Barone and cols. showed that among patients with diabetes living in Brazil more than 59% experienced a high variability in glucose levels during the pandemic, 38.4% postponed their medical appointments, and 59.5% reduced their physical activity (9). In our study, individuals who had greater difficulty managing diabetes were more likely to experience worsening mental health scores during the pandemic. In the opposite, those who perceived an improvement in diabetes control had a lower chance of worsening in this parameter. Our findings also reflect the impact of perceptions regarding diabetes care in mental health *status* of these individuals, as previously described by Lange and Piette (10). This result is alarming as other Brazilian studies have also demonstrated a significant worsening of mental health parameters in patients with diabetes. Alessi and cols. showed that almost 43% of patients with diabetes showed evidence of significant psychological distress during the pandemic, with a greater tendency in those with type 2 diabetes (3). Another Brazilian study found that psychological symptoms were experienced by almost 61% of patients with type 1 diabetes (11).

In this scenario of lifestyle changes and suspension of elective care, having a longer duration of diabetes and using insulin were associated with worsening in self-care scores, which may reflect a profile of patients who would probably benefit most from maintaining medical care in crisis situations. Finally, in our study those patients who followed the social distancing recommendation had a lower risk of self-care deterioration, which may be explained by the convenience of managing diabetes at home during quarantine, and a greater motivation to optimize diabetes care during critical times.

Despite potential limitations, such as using screening-focused questionnaires and a predominantly female sample, our study broadens our understanding of mental health decline predictors in diabetes mellitus patients. It also indicates that certain clinical and socioeconomic characteristics could be beneficial in identifying patients at an increased risk of worsening mental health and self-care, indicating who may require more thorough monitoring in crises.
